# Changes in internalizing and externalizing problems in Dutch children and adolescents receiving outpatient youth care before and during the COVID-19 pandemic

**DOI:** 10.1007/s00787-025-02665-1

**Published:** 2025-02-24

**Authors:** Emma M. Broek, Ronald De Meyer, Rachel van der Rijken, Josjan Zijlmans, Hedy A. van Oers, Michiel A. J. Luijten, Hekmat Alrouh, Arne Popma, Meike Bartels, Robert R. J. M. Vermeiren, Tinca J. C. Polderman, Jacintha M. Tieskens

**Affiliations:** 1 LUMC Curium - Child and Adolescent Psychiatry, Leiden University Medical Center, Leiden, The Netherlands; 2 Praktikon, Nijmegen, The Netherlands; 3 Amsterdam UMC, Child and Adolescent Psychiatry and Psychosocial Care, Amsterdam, The Netherlands; 4 Amsterdam Public Health, Mental Health, Amsterdam, The Netherlands; 5 Amsterdam UMC, Emma Children’s Hospital, Child and Adolescent Psychiatry and Psychosocial Care, Amsterdam, The Netherlands; 6 Amsterdam Public Health, Quality of Care, Amsterdam, The Netherlands; 7 Amsterdam Reproduction and Development, Child Development, Amsterdam, The Netherlands; 8 Amsterdam UMC, Vrije Universiteit Amsterdam, Epidemiology and Data Science, Amsterdam, The Netherlands; 9 Amsterdam Public Health, Methodology and Mental Health, Amsterdam, The Netherlands; 10 Department of Biological Psychology, Vrije Universiteit Amsterdam, Amsterdam, The Netherlands; 11 Levvel, Academic Center for Child and Adolescent Psychiatry, Amsterdam, The Netherlands; 12 Youz, Parnassia Psychiatric Institute, The Hague, The Netherlands; 13 Karakter Child and Adolescent Psychiatry University Centre, Nijmegen, The Netherlands; 14 Department of Child and Adolescent Psychiatry and Accare Child Study Center, University of Groningen, University Medical Center Groningen, Groningen, The Netherlands

**Keywords:** COVID-19, Child and adolescent mental health, Youth care, Longitudinal study

## Abstract

The COVID-19 pandemic significantly impacted the mental health of children and adolescents, but its effects on treatment outcomes in youth care remain unclear. This study examines whether the pandemic influenced treatment outcomes for internalizing and externalizing problems in children receiving outpatient youth care. We analyzed data from three groups of children who received outpatient youth care (*N* = 1090, *M*_*age*_ = 12.85): those treated entirely before the pandemic, those who transitioned into pandemic-related measures during treatment, and those treated entirely during the pandemic. The Child Behavior Checklist was used to assess internalizing and externalizing problems at the start and end of treatment. Repeated measures ANCOVAs and χ^2^ tests of independence were conducted to evaluate changes in problem severity and clinical status, and ANCOVAs were used to compare between-group differences. The results suggest that while overall treatment outcomes were not directly affected by the pandemic, fewer children treated during the pandemic recovered from externalizing problems compared to those treated before the pandemic. Additionally, children treated entirely during the pandemic exhibited higher levels of internalizing and externalizing problems at both the start and end of treatment, and those transitioning into the pandemic also had elevated externalizing problems at both time points. Although the pandemic did not affect the change in problems during treatment, the increased severity of issues at both the start and end of treatment indicates a deterioration in child mental health since the pandemic. Appropriate support, preventive interventions, and regular monitoring of mental health problems may mitigate future impacts.

**Supplementary Information:**

The online version contains supplementary material available at 10.1007/s00787-025-02665-1.

## Background

The COVID-19 pandemic and its restrictive measures severely disrupted people’s lives, particularly those of young people [1–4]. In the Netherlands, children and adolescents (hereafter referred to as children) experienced restrictions such as school and childcare facility closures, lockdowns, curfews, social distancing, and quarantine [5, 6]. Restrictions like social distancing and lockdowns also disrupted treatment provision and delivery for children receiving mental health care. As a response to these restrictions the field of mental healthcare experienced a rapid transition to telehealth [7–9] and mental healthcare workers reported increased psychological problems themselves [10–12]. Mental health professionals also reported overall poorer working conditions as a result of the pandemic [8–12]. These factors may have resulted in poorer treatment outcomes during the COVID-19 pandemic.

Previous findings have shown that children with mental health problems reported deteriorating mental health outcomes over the course of the pandemic [13–16]. Additionally, both cross-sectional and prospective studies with measurements from both before and during the pandemic have indicated that children experiencing psychological or psychiatric problems reported worse mental health during the COVID-19 pandemic compared to before the pandemic. This includes both internalizing and externalizing problems, such as depressive feelings, anxiety, irritability, attention problems, hyperactivity, obsessions or compulsions, and fatigue [17–21]. These studies seem to suggest poorer mental health outcomes in these vulnerable children during the COVID-19 pandemic.

However, none of these studies included longitudinal data obtained in separate groups of children who were treated before and during the COVID-19 pandemic. It is therefore difficult to conclude whether children entered mental health care with elevated problem severity during the pandemic compared to before the pandemic, or whether treatment effects or outcomes were affected by the pandemic resulting in poorer mental health at the end of treatment. Therefore, data from children treated at different moments during the pandemic is needed, both before and after treatment.

This approach has been taken in a recent study in adults treated for mental health problems by de Beurs et al. [22], in which mental health problems were compared between three groups: adults who received treatment entirely before the COVID-19 pandemic, adults who received treatment partially during the COVID-19 pandemic, and adults who received treatment entirely during the COVID-19 pandemic. Results indicated no diminished effectiveness of mental health treatment during the COVID-19 pandemic, as the reduction in symptoms over time between these three groups was found to be the same [22].

In this study we used a similar approach as de Beurs et al. [22] to investigate whether treatment outcomes of children receiving outpatient youth care in the Netherlands was similarly (un)affected during the COVID-19 pandemic. Youth care in the Netherlands is a system of services and support aimed at addressing the mental, emotional, and social needs of children and adolescents up to the age of 18 and their families. The system is designed to help young people facing challenges such as behavioral problems, mental health issues, developmental disorders, and family-related difficulties. The system encompasses a range of services, including preventive care, counseling, outpatient therapy, residential care, and crisis intervention. It aims to support children within their families and communities whenever possible. Collaboration between different professionals, such as psychologists, social workers, and educators, is a key feature of the system, ensuring that children receive comprehensive and coordinated care.

In this study we employed a longitudinal research design in which we compared internalizing and externalizing problems in three groups of children in outpatient youth care settings in the Netherlands: (1) Children who received treatment entirely before the COVID-19 pandemic, (2) children who started their treatment before COVID-19 measures were implemented and completed treatment while the pandemic was still in effect, and (3) children who started and completed treatment entirely during the COVID-19 pandemic. Given that the pandemic was a new experience and there is limited research available in this particular population, an exploratory approach was used. We firstly inspected whether treatment outcomes were affected by the COVID-19 pandemic by testing whether (a) the change in symptoms over time and (b) the clinical status at the end of treatment differed between these groups. Next, we tested whether children entered care with more problems during the COVID-19 pandemic compared to before the pandemic, and finally we tested whether children who were treated during the COVID-19 pandemic left care with more problems than peers treated before the pandemic.

## Methods

### Participants

We used data from children who were treated in Dutch youth care organizations that participate in the Learning Database Youth [23]. These organizations collect and bring together data on the mental health of children receiving youth care. Data was made available anonymously, not reducible to persons or small groups of persons, and therefore, under current European legislation, not considered as personal data. Explicit permission to use the data for scientific research was therefore not necessary. In this study, children between the ages of 8 and 18 years who received outpatient youth care between 2014 and 2022 were included. Data of 13 youth care institutions were used. These centers are situated in northern (45.9%), eastern (34.1%), southern (3.2%), and western (16.8%) parts of the Netherlands. Children and their families received outpatient care for a broad range of problems. Families were primarily referred to outpatient family-oriented treatment due to emotional and behavioral problems of their child related to parental stress. This type of treatment involves a systemic approach to addressing issues within the family dynamic, with a focus on understanding and addressing the interrelated factors within the family system that may contribute to the presenting problems. An overview of the problems of the children that were reported by the parents before entering treatment are depicted in Table 1. Family-oriented (89.91%), individual (2.29%), and other forms of treatment (7.80%) were provided by the institutions. A total of *N* = 1090 caregivers of individual children participated in our study, with 85.50% of the informants being female. In our sample 38.53% of the children were female, and 61.47% were male. The mean age of the children was 12.85 (*SD* = 2.83; range = 8–18) years. The average treatment duration was 241.2 (*SD* = 114.0) days. See Table 1 for further characteristics of the study population and the three groups.

**Table 1:** Descriptive results of the three COVID groups

	COVID group			
	Before pandemic (1)	Transition into pandemic (2)	During pandemic (3)	
Initial sample size (*N*)	668	94	328	

	*M (SD)*	*M (SD)*	*M (SD)*	*F*(*df*_*within*_)^c^	*p*	*Post-hoc*^*h*^
Age at start of treatment	12.84 (2.88)	12.06 (2.80)	13.12 (2.69)	5.13 (1087)	.006	1 > 2^a^; 1 = 3; 2 < 3^a^
Treatment duration (days)	229.5 (101.5)	348.9 (179.1)	233.9 (97.5)	50.22 (1087)	< .001	1 < 2^b^; 1 = 3; 2 > 3^b^

	*N* (%)	*N* (%)	*N* (%)	χ^2^(*df*)	*p*	
Sex (female)	264 (39.52)	31 (32.98)	125 (38.1)	1.52 (2)	.467	
Sex informant (female)	577 (86.38)	77 (81.91)	278 (84.76)	1.54 (2)	.464	
Clinically significant problems at start of treatment						
Both internalizing and externalizing^d^	332 (49.70)	57 (60.64)	196 (59.76)			
Externalizing only^e^	149 (22.31)	18 (19.15)	56 (17.07)			
Internalizing only^f^	68 (10.18)	9 (9.57)	33 (10.06)			
None^g^	119 (17.81)	10 (10.64)	43 (13.11)			

^a^*p* < .05; ^b^*p* < .001; ^c^*df*_*between*_ = 2 for all *F*-tests; ^d^Defined as *T* score ≥ 60 on both the internalizing and externalizing subscales of the Child Behavior Checklist; ^e^Defined as *T* score ≥ 60 on the externalizing and *T* score of < 60 on the internalizing subscales of the Child Behavior Checklist; ^f^Defined as *T* score ≥ 60 on the internalizing and *T* score of < 60 on the externalizing subscales of the Child Behavior Checklist; ^g^Defined as *T* score < 60 on both the internalizing and externalizing subscales of the Child Behavior Checklist; ^h^Independent samples *t*-tests were used to test for specific between-group differences

### Design and procedure

We employed a longitudinal observational design. Data collection is part of the regular treatment in youth care. Caregivers were asked to fill out questionnaires about their children at the start and end of treatment. We exclusively utilized measurements completed by the same informant at both the start and end of treatment and ensured that they were assessed within three months of the treatment’s start- or end date. We used mother ratings (~ 82%), or father ratings if these were not available (~ 14%). If both mother and father ratings were not available, we used reports from other caregivers, including foster parents (~ 2.7%), adoptive parents (~ 0.5%), stepparents (~ 0.5%), and grandparents (~ 0.3%).

### Subgroups used in the analyses

We compared mental health problems between three groups of children in outpatient youth care with different treatment times relative to the pandemic in the Netherlands. The approach is similar to the design used by de Beurs et al. [22]. The first group (“Before pandemic”) consisted of children who started and ended treatment prior to March 16, 2020, when COVID-19 restrictions were first implemented in the Netherlands [5]. The second group (“Transition into pandemic”) consisted of children who started treatment prior to March 16, 2020, and completed treatment after March 16, 2020. Finally, the third group (“During pandemic”) consisted of children who started their treatment after March 16, 2020, and ended their treatment before December 31, 2022.

**Fig. 1:**
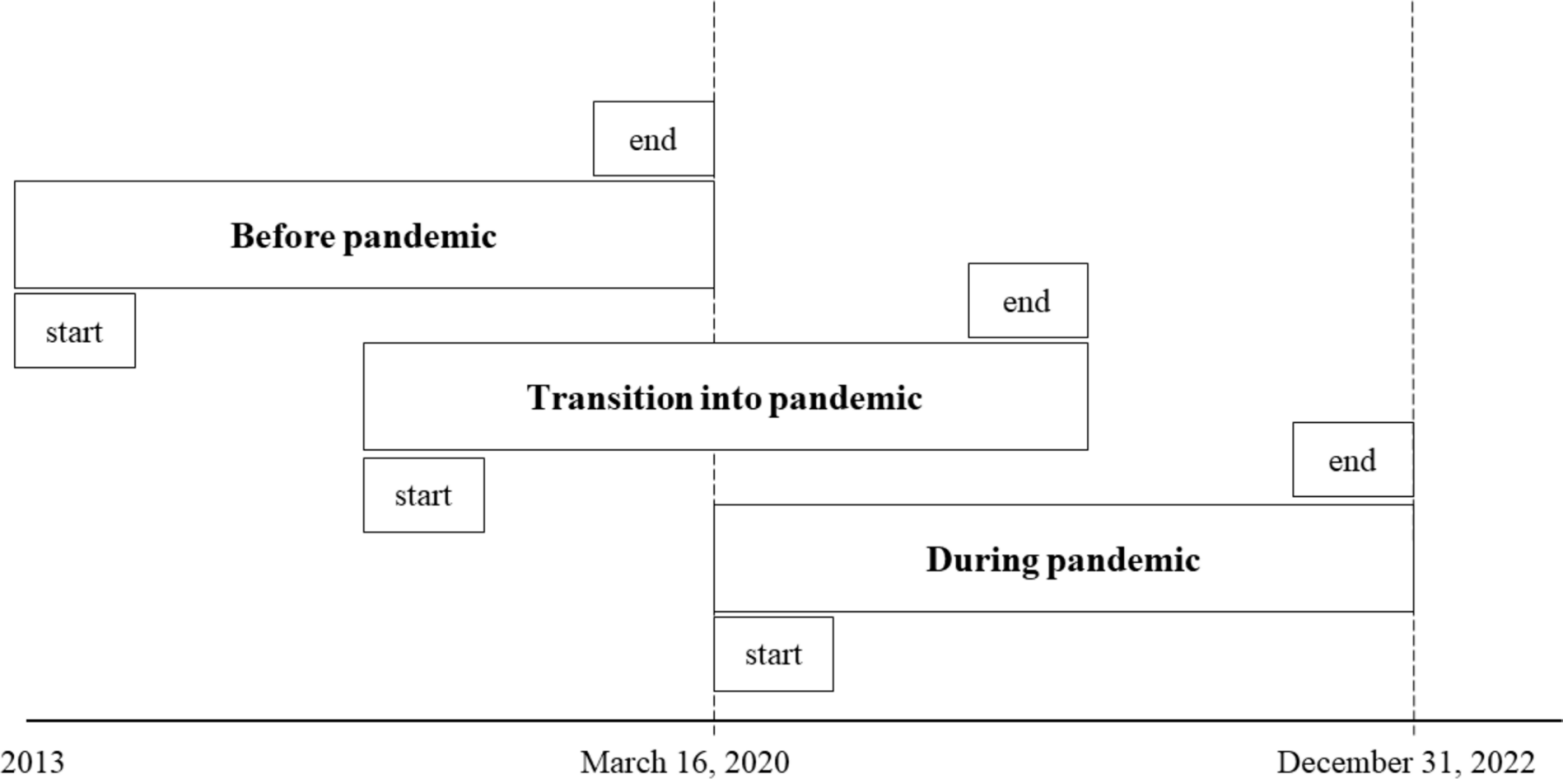
**Subgroups used in analyses.** Graphical representation of groups used in statistical analysis based on start and end of treatment relative to March 16, 2020 (i.e., relative to the COVID-19 pandemic)

Data from the start of treatment was available from *N* = 4,435 caregivers from individual children, and follow-up data was available for *N* = 1,477 children. Children were excluded when they had a treatment duration shorter dan 40 days (short-term treatment) to ensure measurable symptom change. In addition, treatments lasting longer than 1020 days were excluded, as this was the maximum number of treatment days possible in the “During pandemic” group. In addition, we excluded children who started treatment after the end of COVID-19 pandemic measures in the Netherlands (23–03–2022; RIVM [24]). After exclusions a sample of *N* = 1090 caregivers from individual children was available for analysis. See Fig. 1 for a visual overview of the three groups used for statistical analysis.

### Measures

*Internalizing and externalizing problems* The internalizing and externalizing problem scales of the Child Behavior Check List (CBCL; [25]) were filled out by caregivers. Items on the CBCL are rated on a three-point Likert scale reflecting how much a statement applies to a child (0 = “not true”; 1 = “somewhat true”; 2 = “very true”). Missing items were scored as 0. To ensure validity of the questionnaires, children with more than 8 missing items of the 120 total problem items were excluded. No imputation techniques were used.

The internalizing problems scale consists of 13 items about being anxious/depressed (e.g., “Cries a lot”), 8 items about being withdrawn/depressed (e.g., “There is very little he/she enjoys”), and 11 items about somatic complaints (e.g., “Overtired without good reason”). The total internalizing score was calculated by transforming the total sum score of the 32 items into a *T* score ranging from 33 to 100 [25].

The externalizing problems scale consists of 17 items about rule-breaking behavior (e.g., “Drinks alcohol without parents’ approval”) and 18 items about aggressive behavior (e.g., “Destroys his/her own things”). Total externalizing score was calculated by transforming the total sum score of the 35 items into a *T* score ranging from 33 to 100 [25].

*Clinical Status* Two indicators were used to operationalize clinical status at the end of treatment: The Reliable Change Index (RCI), a statistic that determines the magnitude of change of two repeated measures required to be considered statistically reliable [26], and Clinical Significance (CS), the cut-off score to indicate elevated problem scores [27].

We considered the reliable change significant when the RCI > 1.65, which corresponds to *p* < .05. In addition, to decide CS a cut-off score of *T* < 60 (normal range) was used to categorize a client as functional or *T* ≥ 60 (borderline clinical range) as dysfunctional at the end of treatment. When RCI and CS are combined clients can be categorized into four levels of outcome [28]: (1) Recovered (RCI > 1.65 and CS < 60), (2) improved (RCI > 1.65 and CS ≥ 60), (3) no change (no significant RCI), and (4) deteriorated (RCI < − 1.65).

*Control variables* Age of the child at the start of treatment was calculated in years. Sex of both the child and informant was coded as male (0) or female (1). When questionnaires were filled in by both caregivers, sex was treated as female. Treatment duration was calculated in days by taking the difference between the recorded end and start date of treatment.

### Statistical analysis

To test possible differences in background variables between the three groups, we performed several descriptive analyses. One-way ANOVAs (for child’s age at start of treatment and treatment duration) and χ^2^ tests of independence (for child’s and informant’s sex) were performed to test possible differences between the groups. In addition, correlations between study (internalizing and externalizing problems at beginning and end of treatment, and clinical status at end of treatment) and control (child’s age at start of treatment, treatment duration, sex of both the child and informant) variables were inspected.

To test whether treatment outcomes were affected by the COVID-19 pandemic we performed two repeated measures ANCOVAs and two χ^2^ tests of independence. The repeated measures ANCOVAs tested whether the change in internalizing and externalizing problems was similar between the three groups. Internalizing and externalizing problems at the start and end of treatment were included as within-subject, time-varying dependent variables and COVID-19 group was included as between-subject independent factor. Age of the child, sex of both the child and informant, and treatment duration were included as covariates. By inspecting the interaction effect between the time-varying factor (i.e., the change in internalizing and externalizing problems over time) and the between-subject factor (i.e., the three groups) we can determine whether the change in problem severity is different between the three groups. *χ*^*2*^ tests of independence were used to assess between-group differences in clinical status (recovered, improved, no change, deteriorated) at the end of treatment. Two *χ*^*2*^ tests of independence were conducted to test between-group differences in clinical status for internalizing and externalizing problems separately. Post-hoc independent samples proportion tests were used to test specific subgroup differences in clinical status proportions if the main χ^2^ test of independence was found to be significant.

To test whether children entered care with elevated problems during the COVID-19 pandemic compared to before the pandemic two ANCOVAs were used to assess between-group differences in internalizing and externalizing problems at the beginning of treatment. Internalizing or externalizing problems at the start of treatment were included as dependent factors, and group as independent factor. Age of the child, sex of both the child and informant, and treatment duration were included as covariates. Post-hoc *t*-tests were used to test between-group differences.

To test whether children who were treated during the COVID-19 pandemic left care with more problems compared to peers treated before the pandemic two ANCOVAs were executed to assess between-group differences in internalizing and externalizing problems at the end of treatment. Internalizing or externalizing problems at the end of treatment were included as dependent factors, and group as independent factor. Age of the child, sex of both the child and informant, and treatment duration were included as covariates. Post-hoc *t*-tests were used to test between-group differences.

## Results

### Descriptive results

Table 1 presents demographic characteristics of the three groups. The “Before pandemic” group was the largest group (*N* = 668), followed by the “During pandemic” group (*N* = 328), and the “Transition into pandemic” group (*N* = 94). Children in the “Transition into pandemic” group were significantly younger (*p* = .006, *M*_*age*_ = 12.06 (*SD* = 2.80)) and had significantly longer treatment duration (*p* < .001, *M*_*treatment*_ = 348.9 days (*SD* = 179.1)) than children in both the “Before pandemic” group (*M*_*age*_ = 12.84 (*SD* = 2.88); *M*_*treatment*_ = 229.5 days (*SD* = 101.5)) and “During pandemic” group (*M*_*age*_ = 13.12 (*SD* = 2.69); *M*_*treatment*_ = 233.9 days (*SD* = 97.5)). The distribution of sex in referred children or informants did not differ between the three groups. In Online Resource 1 correlations between all study and control variables are depicted.

### Internalizing problems

**Fig. 2:**
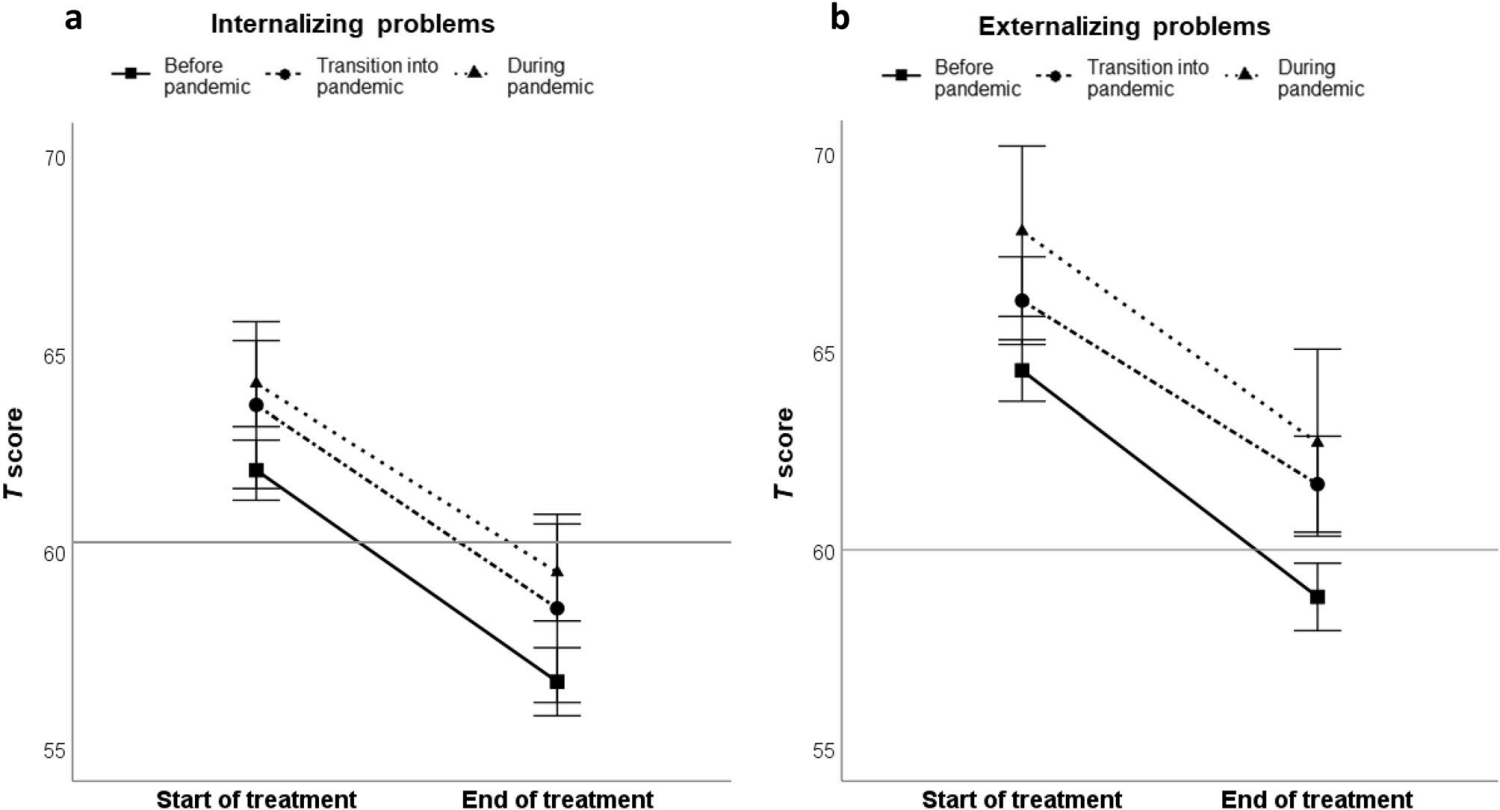
**Change over time of internalizing and externalizing problems.** Change over time of (**a**) internalizing problems and (**b**) externalizing problems (measured with the CBCL) for the three groups relative to the COVID-19 pandemic. The horizontal line at *T* score = 60 shows the cutoff for internalizing and externalizing problems at clinical levels. Internalizing and externalizing problems at start and end of treatment are reported corrected for child’s and informant’s sex, child’s age at start of treatment, and treatment duration

In all groups, there was a significant decrease in internalizing problems (*F*(1,1083) = 8.55, *p* = .004, partial η^2^ < .01). The change in internalizing problems from start to end of treatment did not differ between the three groups (*F*(2,1083) = 0.44, *p* = .64, partial η^2^ < .001). See Fig. 2a for a graphical representation of these results. Clinical status based on internalizing problems did not differ between the three pandemic groups at the end of treatment (*χ*^*2*^(6) = 7.59, *p* = .270, see Table 2). Internalizing problems at the beginning (*F*(2,1083) = 5.57* p* = .004, η^2^ = .01) and at the end (*F*(2,1083) = 6.84, *p* = .001, η^2^ = .01) of treatment were significantly different between the three groups. Post-hoc *t*-tests revealed that internalizing problems in the “During pandemic” group were significantly higher than in the “Before pandemic” group at both the beginning and end of treatment. No other between-group differences were found at either time point (see Table 3).

**Table 2:** Clinical outcome categories between COVID groups

	COVID group					
	Before pandemic (1)	Transition into pandemic (2)	During pandemic (3)			
	*N* (%)	*N* (%)	*N* (%)	χ^2^(*df*)	*p*	
RCI category internalizing problems CBCL				7.59 (6)	.270	
No change	430 (64.37)	67 (71.28)	231 (70.43)			
Improved	28 (4.19)	2 (2.13)	14 (4.27)			
Recovered	179 (26.80)	24 (25.53)	72 (21.95)			
Deteriorated	31 (4.64)	1 (1.06)	11 (3.35)			
RCI category externalizing problems CBCL				12.88 (6)	.045	Post-hoc^b^
No change	415 (62.13)	64 (68.09)	215 (65.55)			1 = 2 = 3
Improved	53 (7.93)	11 (11.70)	41 (12.50)			1 = 2; 2 = 3; 1 < 3^a^
Recovered	177 (26.50)	17 (18.09)	63 (19.21)			1 = 2; 2 = 3; 1 > 3^a^
Deteriorated	23 (3.44)	2 (2.13)	9 (2.74)			1 = 2 = 3

Differences between the three COVID groups in internalizing and externalizing clinical outcome categories based on the Reliable Change Index

*RCI* Reliable Change Index, *CBCL* Child Behavior Checklist

^a^*p* < .05; ^b^Independent-samples proportion tests

**Table 3:** Differences in internalizing and externalizing problem severity between COVID groups

	COVID group					
	Before pandemic (1)	Transition into pandemic (2)	During pandemic (3)			
	*M* *(SD)*	*M* *(SD)*	*M* *(SD)*	*F*(*df*_*within*_)^c^	*p*	*Post-hoc*^*e*^
Internalizing problems start treatment (*T* score)^d^	62.05 (10.26)	63.51 (8.33)	64.25 (10.25)	5.57 (1083)	.004	1 = 2; 1 < 3^a^; 2 = 3
Internalizing problems end treatment (*T* score)^d^	56.70 (11.47)	58.06 (10.53)	59.55 (11.42)	6.84 (1083)	.001	1 = 2; 1 < 3^b^; 2 = 3
Externalizing problems start treatment (*T* score)^d^	64.63 (9.93)	66.89 (9.68)	66.39 (11.34)	6.48 (1083)	.002	1 < 2^a^; 1 < 3^a^; 2 = 3
Externalizing problems end treatment (*T* score)^d^	58.90 (10.94)	61.54 (10.56)	61.81 (12.18)	9.81 (1083)	< .001	1 < 2^a^; 1 < 3^b^; 2 = 3

^a^*p* < .05; ^b^*p* < .001; ^c^*df*_*between*_ = 2 for all *F*-tests; ^d^Internalizing and externalizing problems at start and end of treatment are reported uncorrected for covariates included in this study (child’s and informant’s sex, child’s age at start of treatment, and treatment duration); ^e^Independent samples *t*-tests were used to test for specific between-group differences

### Externalizing problems

In all three groups, there was a significant decrease in externalizing problems (*F*(1,1083) = 13.68, *p* < .001, partial η^2^ < .01). The change in externalizing problems from start to end of treatment did not differ between the three groups (*F*(2,1083) = 1.85, *p* = .158, partial η^2^ = .003). See Fig. 2b for a graphical representation of these results. Clinical status based on externalizing problems differed between the groups at the end of treatment (*χ*^*2*^(6) = 12.88, *p* = .045). Post-hoc testing was subsequently conducted to find specific between-group differences. Post-hoc independent-samples proportion tests showed that fewer children in the “During pandemic” group (19.21%) recovered from externalizing problems than in the “Before pandemic” group (26.50%). However, more children in the “During pandemic” group (12.50%) achieved reliable change than in the “Before pandemic” group (7.93%). No other between-group differences were found to be significant (see Table 2). Externalizing problems at the start (*F*(2,1083) = 6.48, *p* = .002, η^2^ = .01) and at the end (*F*(2,1083) = 8.81, *p* < .001, η^2^ = .02) of treatment were significantly different between the three groups. Post-hoc *t*-tests revealed that problem severity in the “During pandemic” group and in the “Transition into pandemic” group was significantly higher than in the “Before pandemic” group, both at the beginning and end of treatment. No other between-group differences were found to be significant (see Table 3). In addition, externalizing problems at the end of treatment were still at clinical levels for the “During pandemic” and “Transition into pandemic” groups (*T* score  ≥ 60; see Fig. 2b).

### Sensitivity analysis

To gain more clarity regarding possible effects of time prior to the COVID-19 pandemic we conducted sensitivity analyses in which we split the “Before pandemic” group into two groups. The first group (“Before pandemic 2014–2016”) included children who started treatment in 2014, 2015, or 2016 (*N* = 294), while the second group (“Before pandemic 2017–2019”) included children who started treatment in 2017, 2018, or 2019 (*N* = 374). Children who were in the “Before pandemic 2014–2016” group were approximately half a year younger than children in the “Before pandemic 2017–2019” group, but treatment duration and sex distribution were similar in these groups. See Online Resource 2 for further comparisons between these groups.

Similar to the main analyses, there were no differences between the four COVID-19 groups with regard to changes in problems over time. However, although the interpretation of the research findings did not change, some subtle differences were present compared to the initial analyses. After splitting the “Before pandemic” group, the differences in clinical status for externalizing problems between the COVID-19 groups were no longer significant. The proportions in the “Before pandemic 2014–2016” and “Before pandemic 2017–2019” groups were also very similar as before their split in the “Before pandemic” group in the main analyses. In addition, the severity of externalizing problems at the start of treatment was higher in the “Transition into pandemic” and “During pandemic” group compared to the “Before pandemic 2014–2016” group, but not compared to the “Before pandemic 2017–2019” group. See Online Resources 3–5 for the complete results of these sensitivity analyses.

## Discussion

We aimed to investigate if mental health problems and treatment outcomes of children receiving outpatient youth care in the Netherlands were affected by the COVID-19 pandemic. Mental health problems were operationalized as children’s internalizing and externalizing problems rated by parents. We used a longitudinal research design in three groups of children that were treated at different time periods relative to the COVID-19 pandemic: “Before pandemic”, “Transition into pandemic”, and “During pandemic”. We tested group differences in (1) the changes in problem scores and changes in clinical status at the end of treatment; (2) reported problem scores when children entered care, and (3) reported problem scores when children left care.

Using a similar approach as de Beurs et al. [22], who studied a sample of adults who received mental health care, we did not find differences between the three groups of children in the overall change in internalizing or externalizing problems over time. All groups showed comparable reductions in internalizing and externalizing problems after treatment compared to before treatment. However, when we investigated the Reliable Change Index and the Clinical Status of the children, we did find that children who received treatment during the COVID-19 pandemic were less likely to obtain the status “recovered” from their externalizing problems compared to children who received treatment before the COVID-19 pandemic.

To further contextualize these findings we extended the design used by de Beurs et al. [22], by also inspecting the level of problems at the start and end of treatment in the three groups. Here we found that children who entered care during the COVID-19 pandemic experienced more internalizing and externalizing problems compared to peers who entered care before the pandemic. In addition, we found that children who experienced the transition into the COVID-19 pandemic during their treatment already experienced more externalizing problems when they entered care compared to their peers who were treated entirely before the pandemic. This particular finding cannot be ascribed to the pandemic, as it did not play a role when these children entered youth care. However, these findings do confirm previous studies showing declining child mental health prior to the COVID-19 pandemic [29–33]. This trend has been linked to a number of factors. For instance, societal changes such as the increased use of social media have been shown to lead to increased mental distress among youth, including suicidal attempts and ideation [34, 35]. Increased academic pressure has also been associated with increased depression, anxiety, and suicidal ideation among youth [36]. In addition, increased long-term poverty may lead to adverse consequences of child well-being, likely due to the additional stress and hardships a child experiences in the context of poverty [37, 38]. Additionally, family and parental factors such as decreased parental mental health seem to be correlated with increased levels of psychological problems in children, particularly depressive feelings [39, 40]. Finally, the recent awareness efforts for mental health may have led to more reports of mental health problems among children, as they interpret their problems as disruptive at lower levels than in previous decades [41]. In short, there are a number of possible reasons to explain the decrease in child mental health prior to the COVID-19 pandemic. The increased internalizing and externalizing problems in children who entered care during the pandemic in our sample may therefore be a continuation of changes already occurring at a larger societal scale, rather than only being a result of the pandemic on child mental health.

Furthermore, we found that children who entered care during the COVID-19 pandemic concluded treatment with higher internalizing and externalizing problems than their peers who were treated before the pandemic. In addition, children who experienced the transition into the pandemic also showed higher externalizing problems at the end of their treatment compared to peers treated before the pandemic. This is in line with previous findings that showed poorer child mental health during the pandemic compared to before the pandemic [17–21, 42, 43] and is potentially partially due to effects of the pandemic on youth care. A substantial proportion of child mental health care had to transition into telehealth early in the pandemic due to social distancing measures [7–9]. Yet, organizations often encountered difficulties early in this process with licensing, infrastructure, or availability of devices for both clinicians and families receiving care [7–9, 44, 45], which may have disrupted treatment. We did find a substantially longer treatment duration in the transitional group, suggesting that this group needed more time to achieve the same therapeutic outcomes as children treated before or entirely during the pandemic. This may indeed be due to difficulties adjusting to COVID-19 restrictions in treatment. However, due to limited background information on potential changes in treatment protocols, it is possible that treatments were more spread out, meaning children in the “Transition into pandemic” group may have received the same amount of care over a longer period as those treated entirely before or during the pandemic. More long-term detrimental effects of COVID-19 on the mental healthcare sector have also been reported, such as persisting increased psychological problems [10–12] and poorer working conditions [8–12] among mental healthcare workers due to the pandemic.

### Implications for practice

Although all three groups achieved similar reductions in internalizing and externalizing problems over time, our findings do suggest that children treated during the pandemic concluded care with more problems than children who were treated before the pandemic. Children who were treated either partially or entirely during the pandemic on average still score at clinical levels of externalizing problems at the end of treatment, whereas this was not the case in our pre-pandemic sample. While it remains uncertain whether these findings are solely attributable to the COVID-19 pandemic or also reflect pre-existing societal changes in child mental health, they underscore the importance of providing appropriate support for children during such large-scale stressful events like the COVID-19 pandemic as well as during broader societal shifts. Additionally, investing in preventive interventions at both clinical and community levels is crucial to mitigate the negative impacts of these events on vulnerable young children. Regular monitoring of child mental health may also facilitate the early identification of problems before clinical significance is apparent, enabling timely intervention within community settings at a level of care more suited to milder problems.

In addition, preventive strategies ought also to include interventions and guidelines at community levels to prevent the development of such psychological problems. Many social structures for children, such as school and sports activities, were stripped from children during the COVID-19 pandemic. Previous research has shown that during such global disasters children experience feelings of anxiety, worry, and problems with attention and concentration [46]. However, feelings of boredom and loneliness were related to school closures in particular during the COVID-19 pandemic, and previous research has shown that vulnerable children are especially more at risk to develop these feelings, putting them in an even more disadvantaged position [46–48]. These finding highlight the need to balance child mental health and public health considerations when making decisions about lockdown measures. In addition, they also show that when a decision is made to increase lockdown measures, strategies should be in place to help children cope with these possible negative consequences for mental health.

Besides preventive strategies, it is important to enhance the resilience of children through promotional interventions that improve the socio-emotional competencies of children allowing them to cope more adaptively with the harmful effects of traumatic situations such as the COVID-19 pandemic. To do so, focus may be put on improving children’s social-emotional functioning and social networks. Previous research has shown that deficits in social-emotional functioning, such as poor self-control [49] or difficulty regulating emotions [50] are associated with increased levels of stress and poorer stress recovery [51], also during the COVID-19 pandemic [49, 50]. In addition, children who experience more social support, both from their friends [52, 53] and their families [16, 49], experience fewer negative emotional effects of the COVID-19 pandemic. This is supported by previous findings that social support contributes to enhanced resilient psychosocial functioning [54]. Therefore, promoting interventions that strengthen children's social-emotional functioning and social support networks can play a crucial role in fostering their resilience, enabling them to better navigate future adversities and maintain emotional well-being.

Finally, an important finding in this study is that a significant proportion of children receiving youth care did not improve or recover from their internalizing or externalizing problems. These results must be put in the context of a Dutch political system that has shifted focus from specialist services to lower-level care in the mental healthcare sector, including youth care [55]. This shift may have resulted in care that is insufficient to consistently achieve recovery or meaningful improvement in the problems reported in this study. Future policy should prioritize care tailored to the specific needs of children by enhancing access to appropriate services and ensuring specialized support is available when necessary.

### Strengths and limitations

This is the first longitudinal study in which the effects of the COVID-19 pandemic on outpatient youth care were investigated using repeated measures in both children who were treated entirely before the COVID-19 pandemic and children treated during the pandemic. Previous prospective studies that included pre-pandemic measurements often did not include children treated entirely during or entirely before the pandemic, limiting the conclusions that could be drawn from their findings. In addition, many studies in which findings from before and during the pandemic were compared were cross-sectional in nature, and could therefore not examine within-person effects of the pandemic on children. We were able to address these limitations by using both repeated measures and three groups of children treated at different time periods relative to the COVID-19 pandemic. In addition, by including children who experienced the transition into COVID-19 measures, we were also able to further understand how the pandemic affected child mental health and treatment (e.g., treatment duration) independently of changes to child mental health that were already occurring before the pandemic. We also included large samples of children and employed reliable and valid instruments to measure child mental health.

Although our study included a large sample that covered an extensive period, we had limited background information about our sample. Besides treatment duration no information was available regarding adjustments to treatment, such as delays to treatment or how much care transitioned to telehealth. In addition, we had limited demographic information available to assess generalizability or to control for any confounding influences because of the General Data Protection Regulation (GDPR). Conclusions about the generalizability of our sample are further limited as the CBCL is not filled out for every child that enters the youth care institutions that are part of the Learning Database Youth. Although data collection is part of regular treatment in youth care, each institution implements this in their own way. For example, not all institutions use the CBCL to measure problems. In addition, parents may not always fill in the questionnaires that are sent to them. Therefore, selection effects might have influenced our results.

In addition, a limitation of this study is the lack of a multi-informant approach of measuring internalizing and externalizing problems. Although previous research has shown that parents may over- or under-report both internalizing and externalizing problems [56–58], this study only used proxy ratings of child mental health. In particular, parents may over-report externalizing problems and under-report internalizing problems in younger children, whereas they may underreport both internalizing and externalizing problems in adolescents [56–58]. It is therefore a possibility that internalizing and externalizing problems may have been under- or overreported. Moreover, previous research has shown discrepancies between maternal and paternal reports of child mental health [59–61]. In this study 18% of respondents were someone other than the mother, which may have led to different reports of child mental health. However, the proportion of informants did not differ between the COVID-19 groups, and for each included participant problems were measured at the beginning and end of treatment by the same informant, limiting informant effects on the between-group differences found in this study. In addition, previous research has shown that parental ratings of child mental health may also be influenced by factors such as parental mental health [62, 63], which in turn may also be influenced by awareness efforts and therefore be overreported [41]. It is therefore possible that the effects of the COVID-19 pandemic or societal changes on adult mental health may have influenced our findings.

Finally, the group sizes between the three COVID groups varied. To test the robustness and validity of our findings sensitivity analyses were conducted in which the “Before pandemic” group was split in two parts more equal in size to the “During pandemic” group. Although subtle differences were detected, the main findings did not change. This suggests limited influence of the imbalance in group sizes.

### Future directions

Although we covered the entire duration of the pandemic, the possible long-term effects of the pandemic could not be measured based on our study. Future studies should compare children who entered care after the COVID-19 pandemic (i.e., after restriction measures ended) to children treated before and during the pandemic, to study whether children’s mental health problems are returning to pre-pandemic levels. In addition, although our study showed that many children are leaving care with clinical levels of problems, it is unclear how these children are doing now. Future longitudinal studies might examine how these groups of children are currently coping.

In addition, to further understand the effects of the COVID-19 pandemic on treatments, future studies might be conducted regarding specific adjustments to care made as a direct result of pandemic restrictions. For example, further research might investigate the influence of factors such as transitions to telehealth and poorer mental health and working conditions of mental healthcare workers on treatment effects. In addition, factors such as socioeconomic status, treatment dropout, and specific types of mental health problems might be evaluated. This information is necessary to better understand the potential effects of these factors on the mental healthcare and problems of youth during global health-related disasters such as pandemics. As previous research has shown that poor child mental health may lead to lifelong negative consequences such as overall poor health and negative social and economic outcomes [64], it is important to further understand how child mental health might be affected by such disrupting global events, both during these events and afterwards.

Finally, future studies may benefit from adopting a multi-informant approach. As parental ratings of child mental health may over- or underestimate psychological problems dependent on the age of their child [56–58, 65, 66] it is important to include both self- and proxy-reports of mental health in future studies including children. In addition, as preliminary evidence suggests that the COVID-19 pandemic had differential effects on younger and older children [14, 15, 67, 68], future studies in vulnerable children should ensure adequate sample sizes to test these groups separately.

## Conclusions

To conclude, we did not find indications that treatment outcomes were affected by the COVID-19 pandemic. However, we did find elevated internalizing and externalizing problem severity in children treated entirely during the pandemic, and elevated externalizing problem severity in children who started treatment during the pandemic compared to children treated before the pandemic. Our findings indicate that child mental health has deteriorated since the start of the pandemic, possibly as a result of both pre-pandemic trends of deteriorating child mental health and due to the COVID-19 pandemic restrictions specifically. As poor mental health at a young age may predict lifelong negative consequences, it is important to study the long-term development and treatment of child mental health. In addition, preventive and promotional interventions and investments in providing adequate care for children may help prevent and mitigate mental health problems both during and after such large-scale events like the COVID-19 pandemic.

## Supplementary Information

Below is the link to the electronic supplementary material.

Supplementary file1 (DOCX 162 KB)

## Data Availability

All data in the present study are available upon reasonable request to the authors.

## Abbreviations

CBCL: Child Behavior Checklist
RCI: Reliable Change Index
COVID-19: Coronavirus disease 2019
