# Supplementary material for: Changes in internalizing and externalizing problems in Dutch children and adolescents receiving outpatient youth care before and during the COVID-19 pandemic
Source: Eur Child Adolesc Psychiatry. 2025 Feb 24;34(8):2521–33. doi: 10.1007/s00787-025-02665-1 (PMC12396983; doi:10.1007/s00787-025-02665-1)
Supplement: Supplementary file 1 — Supplementary file1 (DOCX 162 KB) [file 787_2025_2665_MOESM1_ESM.docx]

**Supplementary materials**

**Submission title: Changes in internalizing and externalizing problems in Dutch children and adolescents receiving outpatient youth care before and during the COVID-19 pandemic**

**Submitted to: European Child & Adolescent Psychiatry**

Emma M. Broek*, Ronald De Meyer, Rachel van der Rijken, Josjan Zijlmans, Hedy A. van Oers, Michiel A.J. Luijten, Hekmat Alrouh, Arne Popma, Meike Bartels, Robert R.J.M. Vermeiren, Tinca J. C. Polderman, & Jacintha M. Tieskens

*Corresponding author: LUMC Curium - Child and Adolescent Psychiatry, Leiden University Medical Center, Leiden, The Netherlands, [e.m.broek@lumc.nl](mailto:e.m.broek@lumc.nl)

**Online Resource 1 Correlations between study and control variables**

*Correlations between study (internalizing and externalizing problems before and after treatment) and control (child’s age at start of treatment, treatment duration, sex of both the child and informant) variables*

|  | 1 | 2 | 3 | 4 | 5 | 6 | 7 | 8 |
| --- | --- | --- | --- | --- | --- | --- | --- | --- |
| 1. Internalizing problems before treatment (*T* score) | - |  |  |  |  |  |  |  |
| 2. Internalizing problems after treatment (*T* score) | .64^b^ | - |  |  |  |  |  |  |
| 3. Externalizing problems before treatment (*T* score) | .49^b^ | .37^b^ | - |  |  |  |  |  |
| 4. Externalizing problems after treatment (*T* score) | .36^b^ | .61^b^ | .71^b^ | - |  |  |  |  |
| 5. Age at start of treatment | .07^a^ | .11^b^ | .10^b^ | .13^b^ | - |  |  |  |
| 6. Treatment duration (days) | .02 | -.02 | -.08^b^ | -.08^b^ | -.27^b^ | - |  |  |
| 7. Sex of child | .10^b^ | .04 | -.05 | -.05 | -.002 | 0.02 | - |  |
| 8. Sex of informant | .09^b^ | .05 | .12^b^ | .06^a^ | -.01 | -.01 | -.02 | - |

Note: ^a^ *p* <.05; ^b^ *p* <.001

**Online Resource 2 Comparison between “Before pandemic 2014-2016” and “Before pandemic 2017-2019” groups**

*Independent samples t-tests and χ^2^-tests of independence to compare the two different groups created from the “Before pandemic” group in the main analyses.*

|  | Before COVID group |  |  |
| --- | --- | --- | --- |
|  | 2014-2016 | 2017-2019 |  |
| Initial sample size (*N*) | 294 | 374 |  |
|  | *M* (*SD*) | *M* (*SD*) | Independent samples *t*-test |
| Age at start of treatment | 12.51 (2.92) | 13.09 (2.83) | *t*(666) = -2.58, *p* = .010 |
| Treatment duration (days) | 236.5 (103.1) | 224.1 (100.1) | *t*(666) = 1.57, *p* = .118 |
|  | *N* (%) | *N* (%) | χ^2^(*df*) |
| Sex (female) | 120 (40.82) | 144 (38.50) | 0.37 (1), *p* = .544 |
| Sex informant (female) | 251 (85.37) | 326 (87.17) | 0.45 (1), *p* = .503 |
| Clinically significant problems at start of treatment | | | |
| - Both internalizing and externalizing ^a^ | 138 (46.94) | 194 (51.87) |  |
| - Externalizing only ^b^ | 61 (20.75) | 88 (23.53) |  |
| - Internalizing only ^c^ | 36 (12.24) | 32 (8.56) |  |
| - None ^d^ | 59 (20.07) | 60 (16.04) |  |

^a^ Defined as *T* score ≥ 60 on both the internalizing and externalizing subscales of the Child Behavior Checklist; ^b^ Defined as *T* score ≥ 60 on the externalizing and *T* score of < 60 on the internalizing subscales of the Child Behavior Checklist; ^c^ Defined as *T* score ≥ 60 on the internalizing and *T* score of < 60 on the externalizing subscales of the Child Behavior Checklist; ^d^ Defined as *T* score < 60 on both the internalizing externalizing subscales of the Child Behavior Checklist.

**Online Resource 3 Change over time of internalizing and externalizing problems, sensitivity analysis**

*Change over time of (a) internalizing problems and (b) externalizing problems (measured with the CBCL) for the four groups relative to the COVID-19 pandemic. The Before pandemic group from the main analyses has been split in two groups based on the year in which treatment was started. The horizontal line at T score = 60 shows the cutoff for internalizing and externalizing problems at clinical levels.*


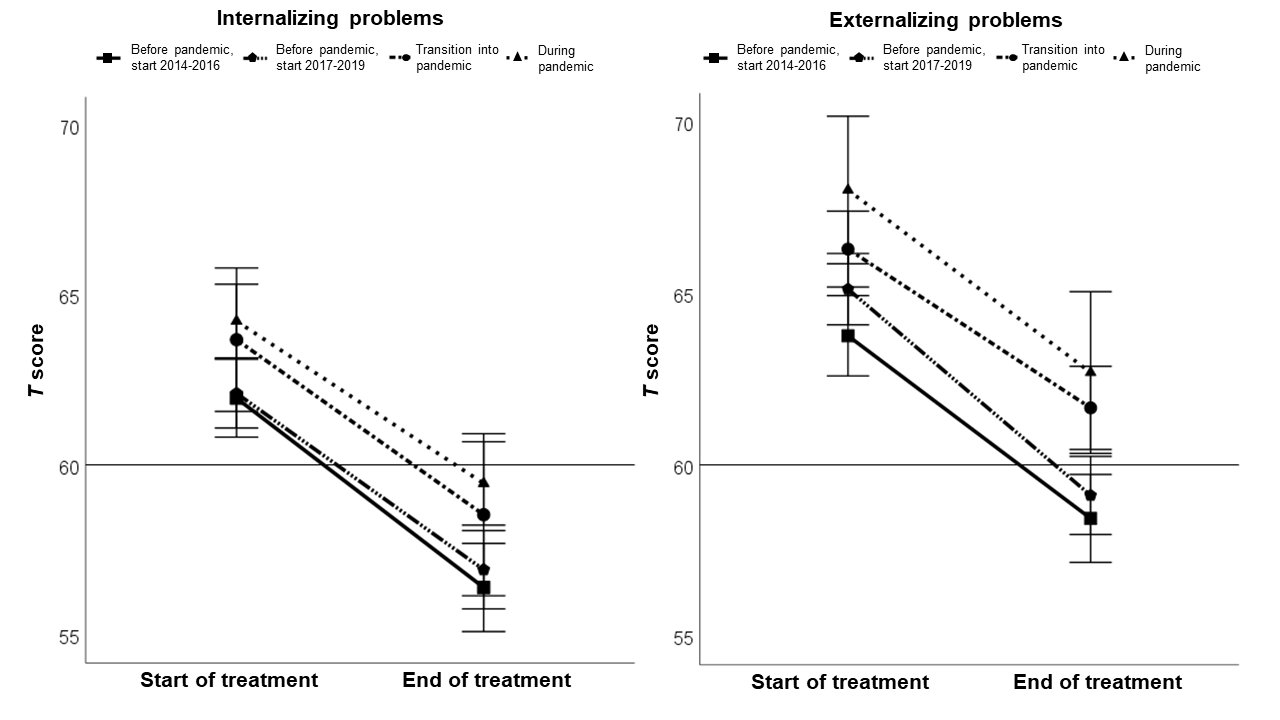


**a**

**b**

Similar to the main analyses we found a significant decrease in internalizing problems for all groups (*F*(1,1082) = 8.83, *p* = .003, partial η^2^ < .008), but the change in internalizing problems from start to end of treatment did not differ between the four groups (*F*(1,1082) = .39, *p* = .76, partial η^2^ < .001). See Online Resource 3a for a graphical representation of these results.

Similar to the main analyses we found a significant decrease in externalizing problems for all groups (*F*(1,1082) = 14.84, *p* < .001, partial η^2^ < .014), but the change in externalizing problems from start to end of treatment did not differ between the four groups (*F*(1,1082) = 1.60, *p* = .19, partial η^2^ < .004). See Online Resource 3b for a graphical representation of these results.

**Online Resource 4** **Clinical outcome categories between COVID groups, sensitivity analysis**

*In these analyses the group of children treated prior to the COVID-19 pandemic has been split into two groups, those who started treatment during the years 2014-2016 and those who started treatment during the years 2017-2019. None of these children started treatment in 2020 or later.*

|  | COVID group | | | |  |  |
| --- | --- | --- | --- | --- | --- | --- |
|  | Before pandemic, start date in 2014-2016 (1) | Before pandemic, start date in 2017-2019 (2) | Transition into pandemic (3) | During pandemic (4) |  |  |
|  | *N (%)* | *N (%)* | *N (%)* | *N (%)* | *χ*^2^ (*df*) | *p* |
| **RCI category internalizing problems CBCL** | | | | |  |  |
| No change | 191 (64.97) | 239 (63.90) | 67 (71.28) | 231 (70.43) | 11.88 (9) | .22 |
| Improved | 17 (5.78) | 11 (2.94) | 2 (2.13) | 14 (4.27) |  |  |
| Recovered | 74 (25.17) | 105 (28.07) | 24 (25.53) | 72 (21.95) |  |  |
| Deteriorated | 12 (4.08) | 19 (5.08) | 1 (1.06) | 11 (3.35) |  |  |
| **RCI category externalizing problems CBCL** | | | | |  |  |
| No change | 185 (62.93) | 230 (61.50) | 64 (68.09) | 215 (65.55) | 13.28 (9) | .150 |
| Improved | 21 (7.14) | 32 (8.56) | 11 (11.70) | 41 (12.50) |  |  |
| Recovered | 78 (26.53) | 99 (26.47) | 17 (18.09) | 63 (19.21) |  |  |
| Deteriorated | 10 (3.40) | 13 (3.48) | 2 (2.13) | 9 (2.74) |  |  |

Acronyms: RCI: Reliable Change Index; CBCL: Child Behavior Checklist

Clinical status based on internalizing problems did not differ between the four pandemic groups at the end of treatment (*χ^2^*(9) = 11.88, *p* = .22).Clinical status based on externalizing problems did no longer differ between the four pandemic groups at the end of treatment (*χ^2^*(9) = 13.28, *p* = .15) after splitting the “Before pandemic” group.

**Online Resource 5** **Differences in internalizing and externalizing problem severity between COVID groups, sensitivity analysis**

*In these analyses the group of children treated prior to the COVID-19 pandemic has been split into two groups, those who started treatment during the years 2014-2016 and those who started treatment during the years 2017-2019. None of these children started treatment in 2020 or later.*

|  | COVID group | | | |  |  |  |
| --- | --- | --- | --- | --- | --- | --- | --- |
|  | Before pandemic, start date in 2014-2016 (1) | Before pandemic, start date in 2017-2019 (2) | Transition into pandemic (3) | During pandemic (4) |  |  |  |
|  | *M (SD)* | *M (SD)* | *M (SD)* | *M (SD)* | *F (df_within_)c* | *p* | *Post-hoc ^e^* |
| Internalizing problems start treatment (*T* score)^d^ | 61.90 (10.16) | 62.17 (10.35) | 63.51 (8.33) | 64.25 (10.25) | 3.82 (1082) | .011 | 1=2, 1=3, 1<4^a^, 2=3, 2<4^a^, 3=4 |
| Internalizing problems end treatment (*T* score)^d^ | 56.26 (11.33) | 57.04 (11.58) | 58.06 (10.53) | 59.55 (11.42) | 4.67 (1082) | .003 | 1=2, 1=3, 1<4^a^, 2=3, 2<4^a^, 3=4 |
| Externalizing problems start treatment (*T* -score)^d^ | 63.68 (10.02) | 65.37 (9.82) | 66.89 (9.68) | 66.39 (11.34) | 5.30 | .001 | 1=2, 1<3^a^, 1<4^a^, 2=3, 2=4, 3=4 |
| Externalizing problems end treatment (*T* score)^d^ | 58.30 (10.71) | 59.37 (11.11) | 61.54 (10.56) | 61.81 (12.18) | 6.73 | <.001 | 1=2, 1<3^a^, 1<4^a^, 2<3^a^, 2<4^a^, 3=4 |

Note: ^a^ *p* <.05; ^b^ *p* <.001; ^c^ *df_between_* = 3 for all *F*-tests; ^d^ Internalizing and externalizing problems at start and end of treatment are reported uncorrected for covariates included in this study (child’s and informant’s sex, child’s age at start of treatment, and treatment duration); ^e^ Independent samples *t*-tests were used to test for specific between-group differences.

Internalizing problems at the beginning (*F*(1,1082) = 3.82, *p* = .011, η^2^ = .01) and at the end (*F*(1,1082) = 4.67, *p* = .003, η^2^ = .01) of treatment were significantly different between the three groups. Post-hoc *t*-tests revealed that internalizing problems in the “During pandemic” group were significantly higher than in the “Before pandemic 2014-2016” and the “Before pandemic 2017-2019” groups at both the beginning and end of treatment. No other between-group differences were found at either time point. This is in line with the main analyses.

Externalizing problems at the beginning (*F*(1,1082) = 5.30, *p* = .001, η^2^ = .014) and at the end (*F*(1,1082) = 6.73, *p* < .001, η^2^ = .018) of treatment were significantly different between the three groups. Post-hoc *t­*-tests revealed that externalizing problems in the “During pandemic” group were significantly higher than in the “Before pandemic 2014-2016” group at both the beginning and end of treatment, and they were higher than in the “Before pandemic 2017-2019” group at the end of treatment. In addition, externalizing problems in the “Transition into pandemic” group were higher than in the “Before pandemic 2014-2016” group at both the beginning and end of treatment, and higher than in the “Before pandemic 2017-2019” group at the end of treatment. No other between-group differences were found at either time point. These results show subtle nuances compared to the main analyses.
